# Multi-View Pareto Optimization for Minimal-Diagnostic-Set Identification of Disease Vectors

**DOI:** 10.3390/insects17040381

**Published:** 2026-04-01

**Authors:** Nuofei Lin, Jingjing Wang, Yixiang Qian, Li Wei, Hongxia Liu, Bo Dai, Songlin Zhuang, Dawei Zhang

**Affiliations:** 1 Engineering Research Center of Optical Instrument and System, The Ministry of Education, Shanghai Key Laboratory of Modern Optical System, University of Shanghai for Science and Technology, Shanghai 200093, China; 233350663@st.usst.edu.cn (N.L.); 242210428@st.usst.edu.cn (Y.Q.); weilioptic@usst.edu.cn (L.W.); slzhuang@usst.edu.cn (S.Z.); dwzhang@usst.edu.cn (D.Z.); 2 Department of Infectious Disease Control, Shanghai Municipal Center for Disease Control and Prevention, Shanghai 201107, China; wangjingjing@scdc.sh.cn

**Keywords:** morphological taxonomy, multi-view learning, automatic species identification, vector surveillance

## Abstract

Accurate identification of disease vectors is crucial for public health, yet distinguishing morphologically similar species demands significant taxonomic expertise and data resources. This study proposes MVP-Net, an AI-driven framework designed to extract a minimal sufficient set of diagnostic anatomical views from multi-view imagery for efficient identification. The framework was evaluated on regionally collected datasets of Calyptratae (8 views) and Culicidae (11 views) from routine surveillance in Shanghai. Under all-view fusion, MVP-Net achieved Top-1 accuracies of 87.04% for Calyptratae and 100% for Culicidae. After Pareto-based view optimization, the required input was reduced to 5 views for Calyptratae and 2 views for Culicidae, lowering computational cost by 37.49% and 81.82%, respectively, while retaining comparable classification performance (86.11% for the recommended Calyptratae configuration and 100% for the recommended Culicidae configuration). These results show that MVP-Net can reduce view redundancy while preserving comparable identification performance within the current Shanghai surveillance setting, providing a practical approach for optimizing regional multi-view auxiliary identification workflows.

## Simple Summary

Accurate identification of disease vectors such as mosquitoes and flies is essential for preventing vector-borne disease outbreaks. However, distinguishing morphologically similar species is challenging and typically requires expert examination of fine diagnostic traits. Existing AI approaches often depend on large, multi-perspective image datasets, which are resource-intensive to acquire. To address this, we developed MVP-Net, an intelligent system that maintains high accuracy with limited data by learning a set of key anatomical views. Using regionally collected fly and mosquito datasets from routine surveillance in Shanghai, the model retained comparable classification performance using only 5 and 2 views for flies and mosquitoes, respectively. This approach reduces image acquisition effort and computational cost and may support regional auxiliary identification workflows.

## 1. Introduction

The escalating global burden of vector-borne diseases, driven by rapid urbanization and climate variability, has created an imperative for efficient and scalable disease vector surveillance [1,2]. Across the disciplines of public health, medicine, and forensic entomology, the precise identification of Diptera, particularly species within the families Culicidae (mosquitoes) and Calyptratae (flies), is paramount for effective vector control and epidemiological modeling [3,4,5]. However, taxonomic identification at the species level remains a bottleneck. It relies on expert examination of minute, highly localized morphological structures, such as wing venation patterns, thoracic setae arrangement (chaetotaxy), and leg banding [6,7]. As the global shortage of trained taxonomists constrains manual processing, computer vision empowered by deep learning (DL) has emerged as a transformative tool to bridge the gap between biological data acquisition and taxonomic classification [1,8].

Despite the success of convolutional neural networks (CNNs) [9] and Vision Transformers (ViTs) [10] in general object recognition, insect species identification remains a challenging fine-grained visual classification (FGVC) task [11,12]. A substantial body of research has focused on identifying insects from field images or automated light traps [13,14,15,16]. While effective for population monitoring, these in-the-wild approaches often rely on low-resolution global features that are susceptible to background noise [17]. They frequently fail to capture the subtle diagnostic traits prioritized by entomologists, which are crucial for distinguishing sibling species (e.g., *Lucilia sericata* and *Lucilia cuprina*) [18,19]. Recent studies utilizing high-definition lab imagery have demonstrated that focusing on specific body parts (e.g., wings) can significantly outperform whole-body classification, as rigid structures provide stable features unaffected by abdominal distortion [20,21].

To emulate the expert taxonomist’s multi-perspective examination, advanced imaging setups increasingly adopt multi-view strategies, capturing specimens from dorsal, lateral, and ventral angles to overcome occlusion [22,23]. However, simply fusing features from all available views introduces significant data redundancy and computational overhead, which is prohibitive for real-time deployment [24]. Furthermore, not all morphological views contribute equally to discrimination; for instance, the dorsal thorax is critical for distinguishing Aedes species, while wing venation is definitive for *Culex* [21]. Indiscriminate fusion of irrelevant views can lead to negative transfer, where noise dilutes the signal from diagnostic traits [25,26]. Therefore, a central challenge in modern computational entomology is to transition from “processing everything” to “learning what matters.”

Recent advancements in differentiable programming and multi-objective optimization (MOO) offer a pathway to address this efficiency–accuracy trade-off. Techniques such as differentiable Top-*k* selection and optimal transport allow neural networks to learn to rank and select the most informative image patches or views in an end-to-end manner [27,28,29]. Moreover, the selection of optimal feature subsets can be rigorously modeled using Pareto optimization, which seeks to identify a set of “Pareto-optimal” solutions where classification accuracy is maximized while computational cost is minimized [30,31]. This paradigm enables the discovery of efficient architectures that are both accurate and lightweight, filtering out redundant information effectively [32].

To address this problem, we propose MVP-Net (Multi-View Pareto Network), a multi-view identification framework designed to select compact sets of diagnostically informative anatomical parts from multi-view image data. The framework was evaluated on two datasets collected through approximately one year of routine surveillance conducted by the Shanghai CDC system, covering the major mosquito and fly taxa under local routine monitoring. These datasets include a primary Calyptratae dataset comprising 12 species and 512 specimens, and an auxiliary Culicidae dataset comprising 5 species and 363 specimens. Under all-view fusion, MVP-Net achieved Top-1 accuracies of 87.04% for Calyptratae, exceeding the best single-view baseline by 3.71%, and 100% for Culicidae. By integrating a Hard-Concrete gating mechanism with Pareto optimization, the required number of input views was reduced from 8 to 5 for Calyptratae and from 11 to 2 for Culicidae, corresponding to reductions in computational cost of 37.49% and 81.82%, respectively, while maintaining comparable classification performance. These results suggest that multi-view morphological identification can be made more efficient through task-specific view selection, supporting the application of the proposed framework for regional auxiliary identification of vector insects.

## 2. Materials and Methods

### 2.1. Dataset Acquisition and Problem Formulation

To validate the proposed multi-view identification framework, two distinct fine-grained datasets were constructed to represent different taxonomic challenges. The primary dataset serves as the core case study, focusing on Calyptratae, while an auxiliary dataset was compiled for Culicidae to examine whether the same framework can be applied to a second vector group under a comparable imaging and analysis pipeline.

**Figure 1:**
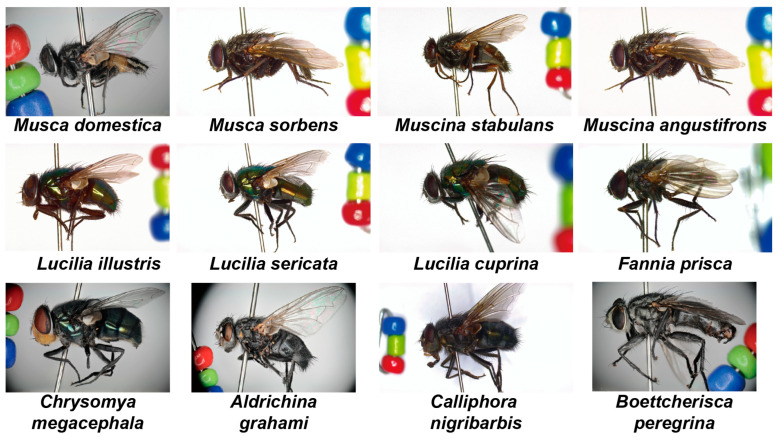
Representative images of the Calyptratae. The dataset includes 12 species of Calyptratae.

All specimens were obtained through routine vector surveillance conducted across various administrative districts of Shanghai, China, with geographic and temporal coverage summarized in Table A1 and Table A4. The procedures for specimen collection, euthanasia, and preparation followed conventional practices employed in CDC-based vector surveillance. Fly specimens were collected either directly using sweep nets or through cage trapping, whereas mosquito specimens were primarily collected using mosquito aspirators. Following collection, live specimens were euthanized with ether or ammonia water and immediately prepared as pinned specimens. In accordance with routine surveillance specimen preparation practices, each pin was inserted vertically through the right side of the mesonotum. Only fresh and intact specimens were retained for imaging and analysis; damaged specimens were discarded during preparation. The prepared specimens were stored in dry specimen boxes and photographed as soon as possible after collection, typically within three months. The species included in the two datasets were selected from the Culicidae and Calyptratae taxa that are common in Shanghai and relevant to routine local surveillance (see Figure 1 and Figure A4 for representative examples).

**Figure 2:**
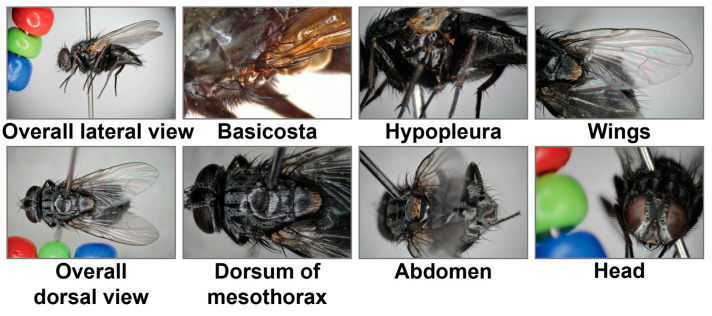
Representative diagnostic morphological structures captured for the primary fly dataset. *Aldrichina grahami* is shown as an example illustrating the 8 targeted morphological views.

Prior to digitization, specimen identification and image annotation were carried out through a collaborative workflow. Experienced taxonomic experts first defined the diagnostic morphological structures to be photographed, based on practical identification experience and relevant taxonomic references [33,34]. For the Calyptratae dataset, eight diagnostic morphological structures, including the basicosta and hypopleura, were selected (Figure 2). Frontline vector surveillance personnel, who had received basic training in locating the required anatomical parts, then acquired images according to the predefined diagnostic views, using the routine microscopic equipment and imaging settings available in the respective regional surveillance units (Table A3). Species identification was primarily performed by two taxonomic experts. In cases where their identifications were inconsistent, an additional taxonomic expert was invited to review the specimen and provide the final determination. This process yielded a total of 512 verified specimens representing 12 species across eight genera. These were partitioned into 404 training samples and 108 validation samples (approximately 4:1 ratio), with the detailed species-wise distribution provided in Table A2.

For the auxiliary Culicidae dataset, 363 specimens spanning 5 species were compiled. In this dataset, 11 specific anatomical parts were defined (Figure A4). These were partitioned into 289 training and 74 validation samples, with the specific species-wise distribution detailed in Table A5.

### 2.2. MVP-Net Architecture and Learning Strategy

To reconcile the critical trade-off between identification accuracy and computational efficiency, MVP-Net is proposed as a unified framework that integrates feature extraction, multi-view synthesis, and differentiable subset selection. Implementation was carried out in Python (version 3.10.19) using the PyTorch (version 2.9.0+cu126) library, with all model training and performance evaluations performed on a workstation equipped with an Intel Core i9-9940X CPU (3.30 GHz), 32 GB of RAM, and an NVIDIA GeForce RTX 4090 GPU.

#### 2.2.1. Feature Extraction and Transformer-Based Fusion

Discriminative representations are first extracted from each independent morphological view using a shared-weight encoder. A ConvNeXt-V2-Pico backbone [35], initialized with ImageNet [36] weights, is employed as the feature extractor, followed by an ML-Decoder head [37]. The ML-Decoder utilizes learnable queries to aggregate spatial features into a compact view-embedding vector, denoted as *vᵢ*.

To preserve the structural context of anatomical parts, a learnable view-specific positional encoding (*PEᵢ*) is injected into the extracted features before fusion:

(1) $$e_{i}=v_{i}+PE_{i}$$

where *eᵢ* serves as the input token for the subsequent fusion module. These tokens are processed via a Transformer-based fusion module utilizing multi-head self-attention [38] with 8 heads. This mechanism allows the model to dynamically assign attention weights to local features based on their relevance to the global classification task, effectively handling occlusion and view-specific noise.

**Figure A1:**
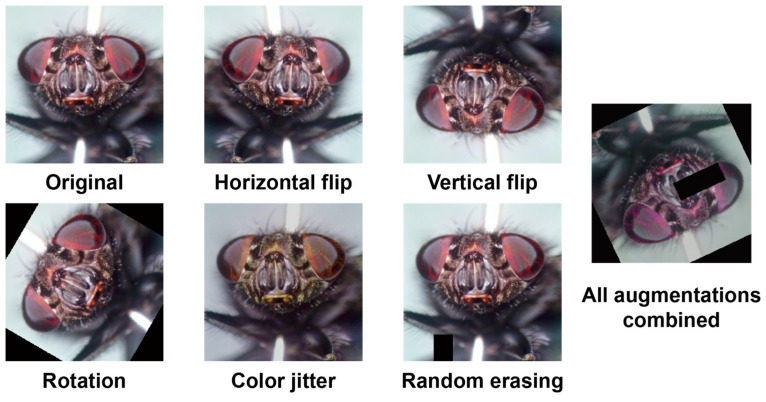
Data augmentation examples generated from a head image of *Aldrichina grahami*.

**Figure A2:**
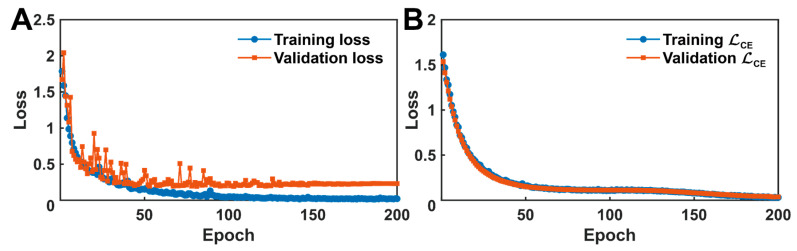
Learning curves on the Calyptratae dataset. (**A**) Training and validation loss curves for the single-view feature extraction stage (Stage 1). (**B**) Cross-entropy loss ($L_{CE}$) curves for the multi-view fusion stage (Stage 2). Note: While the fusion network is optimized using a compound objective function ($L_{total}$), only the classification component ($L_{CE}$) is visualized here to explicitly demonstrate the convergence of the discriminative task.

The backbone is fine-tuned using the AdamW optimizer [39] with an initial learning rate of 1 × 10^−6^ and a weight decay of 5 × 10^−3^, following a cosine annealing warm restart schedule. All input images were resized to 224 × 224 pixels prior to being fed into the network during training, whereas in practical applications, the neural network can adapt to images of arbitrary size. During training, data augmentation techniques, including random flipping, rotation (±60°), color jittering, and random erasing, were applied to enhance robustness against image variability (Figure A1). The learning curves for the single-view feature extraction stage and the multi-view fusion stage are shown in Figure A2, indicating stable convergence throughout the training process.

#### 2.2.2. Differentiable View Selection via Pareto Optimization

To autonomously identify the minimal sufficient set of views, the model aims to minimize the number of active inputs, which corresponds to optimizing the *L*_0_ norm. Since the standard *L*_0_ norm is non-differentiable, a continuous relaxation based on the Hard-Concrete distribution [40] is implemented.

For each view *i*, a binary gate variable *zᵢ* ∈ {0, 1} is sampled from a rectified distribution parameterized by a learnable logit *αᵢ*. To enable gradient-based optimization, the reparameterization trick is applied:

(2) $$z_{i}=\mathrm{clip}\left(\sigma \left(\frac{\log u-\log\left(1-u\right)+\alpha_{i}}{\beta }\right)\cdot \left(\zeta -\gamma \right)+\gamma ,\;0,\;1\right)$$

where $u∼U\left(0,1\right)$ is a uniform random noise, $\sigma \left(\cdot \right)$ is the sigmoid function, and β is the temperature parameter annealed from 0.67 to 0.1 during training. The stretching parameters are set to ζ= 1.1 and γ= −0.1 to allow for exact zero/one sampling. The gated output is then computed as $z_{i}⊙e_{i}$.

The network is trained for 300 epochs using a compound objective function:

(3) $$L_{total}=L_{CE}+\lambda_{contrast}L_{contrast}+\lambda_{sparsity}L_{L_{0}}$$

where

(1) *L*_CE_ is the standard cross-entropy loss.
(2) *L*_contrast_ denotes the supervised contrastive loss [41] with a temperature parameter *τ* = 0.1. The weighting coefficient *λ*_contrast_ is set to 0.2 to enhance the separation of sibling species.
(3) $L_{L_{0}}$ represents the expected *L*_0_ norm (computational cost), calculated as the sum of the gate probabilities:

(4) $$L_{L_{0}}=\sum_{i=1}^{N}\sigma \left(\alpha_{i}-\beta \log\frac{-\gamma }{\zeta }\right)$$

To construct the Pareto frontier, a grid search is performed over *λ*_sparsity_ ∈ {0.1, 0.2, …, 0.5}. The optimal configuration is then selected to minimize the view count while maintaining accuracy within a 1% margin of the all-view baseline.

### 2.3. Software Implementation for Offline Identification

**Figure A3:**
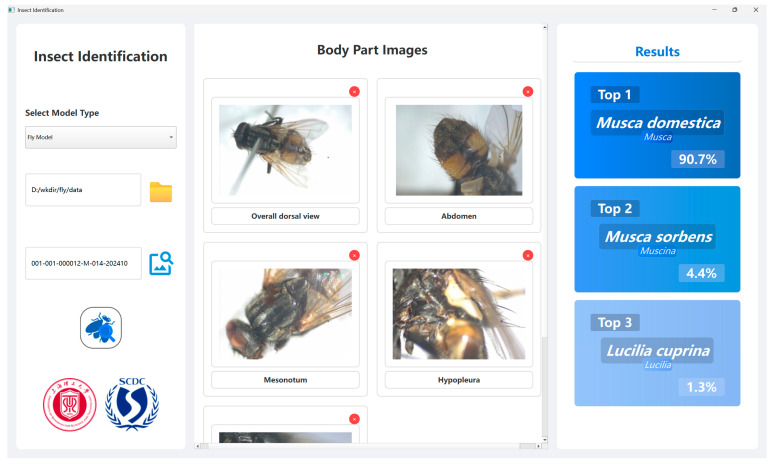
Graphical user interface of the offline multi-view identification software. The software provides a centralized dashboard for specimen management and offline inference in resource-constrained environments. Users can select model types, configure storage paths, and upload images corresponding to the Pareto-optimized morphological views. Input images are automatically processed by the local ConvNeXt-Transformer engine to display the top-3 predicted species with confidence scores and generate standardized CSV identification logs.

**Figure A4:**
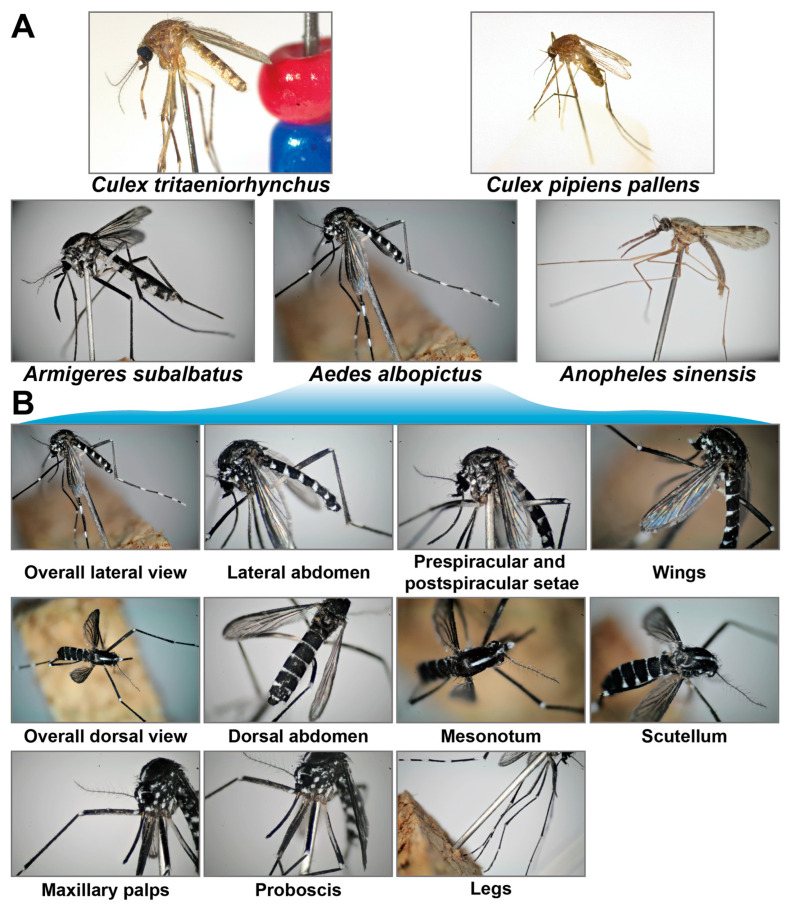
Representative images of the Culicidae (**A**) and diagnostic morphological structures (**B**).

To facilitate the application of the proposed framework in routine use scenarios, a customized desktop application was developed in Python to integrate the Pareto-optimized multi-view identification pipeline. The graphical user interface (GUI) was built using PySide6 (version 6.6.1), providing functionalities for specimen management and image uploading (Figure A3). The software was designed for offline local operation, allowing users to upload images corresponding to the selected morphological views described in Section 2.2. These images are automatically preprocessed by the backend and subsequently passed to the local ConvNeXt–Transformer network for inference. To enable convenient use on standard laboratory computers, the software environment, trained models, and required dependencies were packaged as a standalone executable (.exe) for the Windows 10/11 operating system using PyInstaller (version 6.12.0).

Upon completion of the analysis, the software displays the top-3 predicted species along with their respective confidence scores on the interface to assist decision-making. Comprehensive identification logs, encompassing the specimen identifier and file paths of the specific morphological views, are automatically generated and exported as comma-separated values (CSV) documents.

## 3. Results

### 3.1. Performance Benchmarking: Single-View vs. Multi-View Fusion

**Figure 3:**
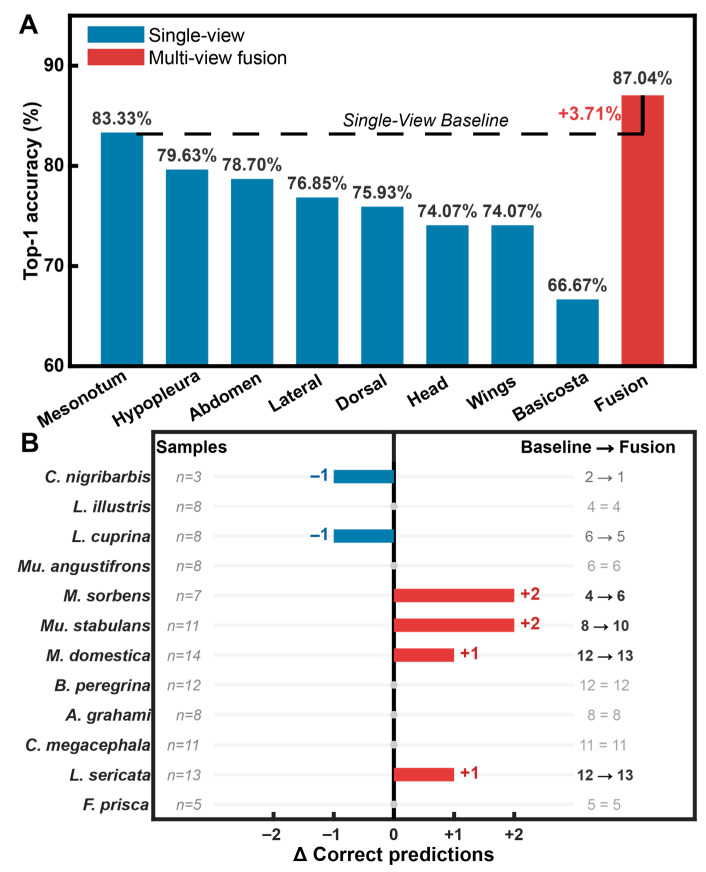
Comparison of single-view classification performance and multi-view fusion. (**A**) Top-1 accuracy obtained from individual morphological views and the multi-view fusion model. (**B**) Species-wise change in the number of correctly classified samples (Δ), with *n* denoting the number of validation samples for each species.

Systematic evaluation on the 12-species Calyptratae validation set demonstrates a consistent performance advantage of multi-view fusion over individual morphological recognition. Among the eight independent views, the mesonotum achieves the highest individual classification accuracy of 83.33% (Figure 3A), serving as the single-view baseline. The Transformer-based fusion model, synthesizing features from all eight views, attains a Top-1 accuracy of 87.04%. This represents an absolute improvement of 3.71 percentage points over the strongest single-view result, confirming that cross-view morphological synthesis is essential for capturing discriminative traits that are otherwise occluded in isolated perspectives.

Species-wise analysis indicates that the impact of multi-view fusion is heterogeneous across different taxa (Figure 3B). For specific disease vectors such as *Musca sorbens* and *Muscina stabulans*, the fusion strategy yields a net gain of two correctly identified samples each, resolving ambiguities inherent in dorsal-only observations. However, evidence of negative interference is also observed in certain categories; for instance, in the cases of *Lucilia cuprina* and *Calliphora nigribarbis*, the fusion of all eight views results in a marginal decrease of one correctly predicted sample compared to the mesonotum baseline. This suggests that indiscriminate feature stacking can introduce localized informational redundancy, which potentially dilutes the diagnostic signals of specific anatomical structures.

Furthermore, the requirement for high-precision multi-view classification on resource-constrained hardware led to the selection of the ConvNeXt-V2-Pico backbone (≈9.1 M parameters). This weight-sharing configuration is intentional, it prunes unnecessary parameter growth across multiple inputs and redirects the available computational capacity toward the Transformer fusion layer to model complex dependencies. Consequently, the architecture navigates these relationships at a cost of just 1.41 GFLOPs per view. Ultimately, such a design ensures that robust taxonomic identification is achieved without compromising inference speed, so as to facilitate high-performance deployment on resource-constrained devices with strict power and memory limitations.

### 3.2. Identification of Pareto-Optimal Subsets and Efficiency Gains

**Figure 4:**
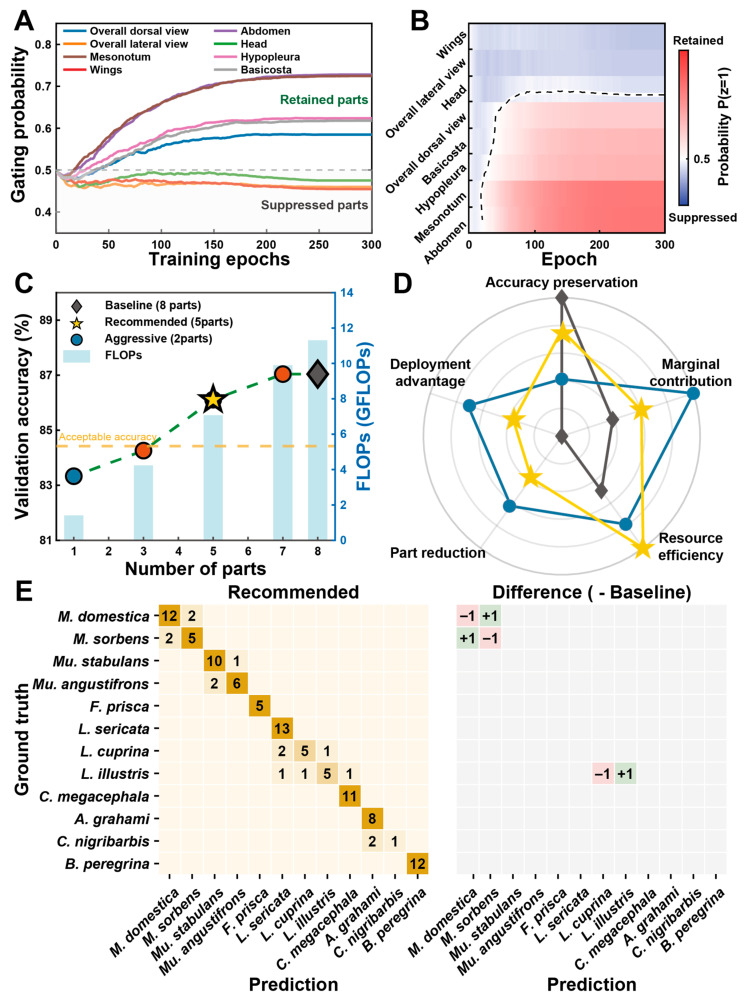
Performance analysis and Pareto-optimized part selection. (**A**,**B**) Evolution of gating probabilities (**A**) and selection dynamics (**B**) for eight morphological views over 300 training epochs under the lambda threshold corresponding to the recommended configuration, where *P*(*z* = 1) represents the retention probability of each view and the dashed line indicates the selection threshold under this lambda constraint. (**C**) Trade-off between validation accuracy, number of selected parts (1 to 8), and computational cost (FLOPs), with the asterisk marking the optimized configuration. (**D**) Radar chart evaluating multi-dimensional performance metrics across different selection strategies. (**E**) Confusion matrix for the recommended 5-view configuration (left), and the species-wise change in the number of correctly classified samples relative to the all-view baseline (right).

The evolution of gating probabilities during the 300-epoch training trajectory illustrates the model’s ability to autonomously prioritize a minimal sufficient set of diagnostic parts (Figure 4A). Anatomical views such as the mesonotum, abdomen, and hypopleura exhibit rapidly increasing retention probabilities, converging toward high-priority status. Conversely, structures including the head, wings, and basicosta are consistently suppressed (Figure 4B), reflecting their lower informational density for fine-grained differentiation within this taxonomic scope. This divergence demonstrates that the differentiable gating mechanism successfully identifies the most discriminative feature combinations while discarding redundant views.

The relationship between the number of views, identification accuracy, and computational overhead is characterized by the Pareto frontier (Figure 4C, Table 1). While the all-view configuration achieves the highest accuracy (87.04%) with a computational cost of 11.31 GFLOPs, the recommended configuration (5-part) identified at lambda λ = 0.4 represents the optimal efficiency-to-performance saturation point. This configuration achieves 86.11% accuracy, maintaining the performance drop within a critical 1% margin (0.93%) relative to the all-view baseline. This recommended configuration is prioritized over the aggressive configuration (3-part) because the latter causes accuracy to drop to 84.26%, representing a 2.78% loss that falls below the predefined threshold for acceptable accuracy.

The superiority of the recommended strategy is further validated by the radar chart (Figure 4D) and Table 1. The recommended configuration represents a strategic peak where the marginal accuracy gain from adding further views (6 through 8) is negligible compared to the 37.49% reduction in theoretical computational cost achieved through part pruning (from 11.31 G to 7.07 G). Species-level confusion analysis (Figure 4E) demonstrates that this optimized subset preserves core diagnostic features, with accuracy losses confined to a single misclassification between the sibling species *M. domestica* and *M. sorbens*. In specific cases like *Lucilia illustris*, the recommended model actually exhibits a marginal improvement (+1 sample) relative to the baseline, suggesting that removing redundant inputs can occasionally mitigate localized classification interference.

To test real-world performance, the identification software (Figure A3) was run on a basic laptop with an Intel Core i7-5500U CPU (2.4 GHz) and 8 GB of RAM. Even without a dedicated GPU, the system achieved real-time inference. This indicates that the lower computational cost (FLOPs) allows the model to work efficiently on common hardware for field use.

### 3.3. Performance Evaluation on the Culicidae Dataset

**Figure 5:**
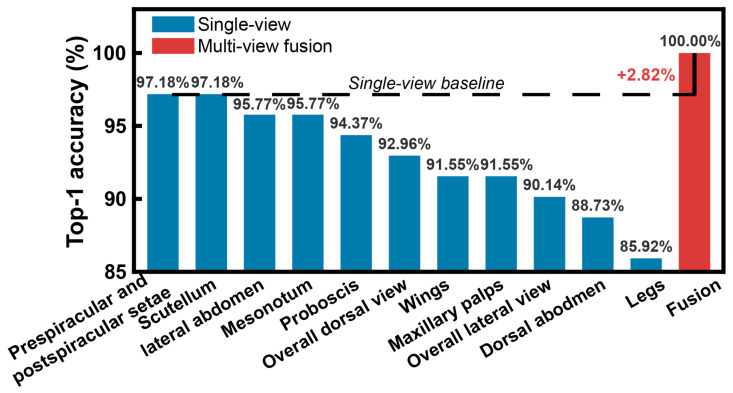
Comparison of single-view classification performance and multi-view fusion.

The framework was further evaluated on the Culicidae dataset to verify its performance across different disease vector taxa. Baseline benchmarking of eleven individual morphological views (Figure 5) shows that the pre-spiracular and post-spiracular setae provides the highest single-view accuracy at 97.18%. In comparison, the integrated multi-view fusion achieves a 100% identification accuracy, representing a performance gain of +2.82%.

**Figure 6:**
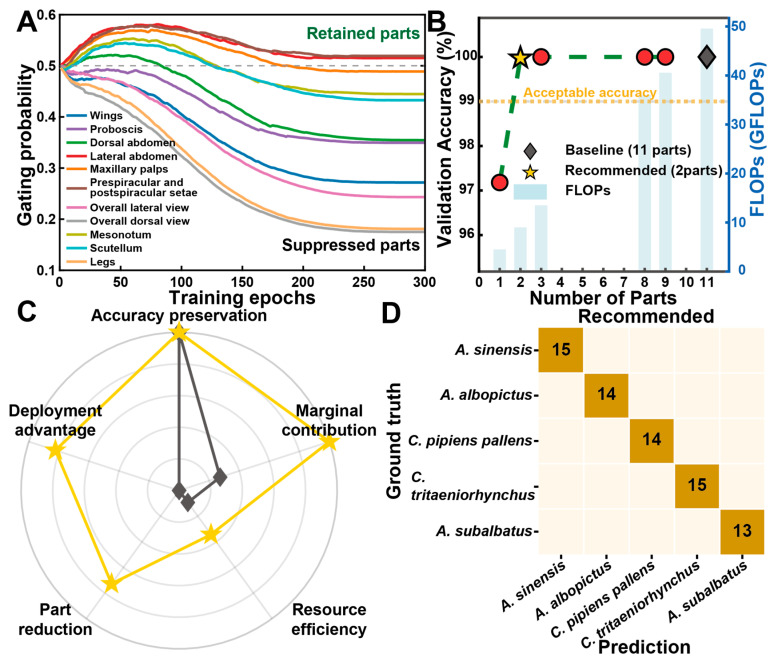
Pareto-optimized part selection for the Culicidae dataset. (**A**) Evolution of gating probabilities for 11 morphological views under the recommended λ configuration. (**B**) Trade-off between validation accuracy, number of selected parts, and computational cost (FLOPs). (**C**) Radar chart of multi-dimensional performance metrics for the recommended 2-part configuration. (**D**) Confusion matrix for the recommended configuration.

Under the recommended λ configuration, the gating mechanism identifies an optimized subset of diagnostic parts (Figure 6A). The selection process consistently prioritizes the prespiracular and post-spiracular setae and lateral abdomen, while suppressing redundant views such as the wings and legs. The resulting 2-part configuration (Figure 6B) maintains the 100% accuracy ceiling while reducing theoretical computational costs (FLOPs) by 81.82% relative to the 11-view baseline (Table 2). Multi-dimensional evaluation (Figure 6C) confirms that this optimized subset achieves an ideal balance between classification precision and resource efficiency.

The confusion matrix (Figure 6D) confirms that the 2-view model achieves absolute precision across all 5 species. These results indicate that the framework can autonomously determine efficient diagnostic part sets for diverse vector groups while maintaining high classification performance.

## 4. Discussion

### 4.1. AI Part Selection and Morphological Consistency

The identification of a minimal sufficient set through MVP-Net suggests that accurate taxonomic differentiation may be achieved without exhaustive morphological coverage, provided that informative diagnostic structures are retained. For Calyptratae, the model consistently prioritizes the mesonotum, abdomen, and hypopleura, which matches the diagnostic characters traditionally used in taxonomic keys. The mesonotum is particularly important as it integrates core diagnostic features, including coloration (e.g., metallic luster), chaetotaxy patterns (such as the arrangement and size of setae), and thoracic markings (e.g., characteristic longitudinal stripes), which collectively serve as the foundation for fly classification. This correspondence indicates that the differentiable gating mechanism is able to select anatomically meaningful views and provides a degree of biological interpretability for the reduced-view configuration.

### 4.2. Task Dependence and Regional Applicability of Minimal View Sets

The samples used in this study were obtained from approximately one year of routine surveillance conducted by the Shanghai CDC system and encompass the major mosquito and fly taxa under routine monitoring. In the 12-species Calyptratae task, the 8-view configuration achieved an accuracy of 87.04%, while reducing the input to 5 views preserved performance within a margin of 0.93%. In contrast, further reduction to 3 views resulted in a decrease in accuracy to 84.26%. These findings suggest that, within the current dataset context, view reduction for Calyptratae is subject to a practical lower threshold. Accordingly, the objective of this framework is not to minimize the number of views at any cost, but rather to identify a compact subset that maintains performance comparable to that of the all-view configuration. Under this premise, the 5-view configuration appears to offer a balanced solution, retaining most of the discriminative information while reducing computational cost by 37.49%.

For the Culicidae dataset, 100% accuracy was achieved using only two views, and the included specimens cover the major mosquito taxa routinely monitored by the Shanghai CDC system. Within the current Shanghai surveillance context, this two-view configuration appears sufficient for identifying the major mosquito taxa under routine monitoring.

These findings indicate that the optimal subset of diagnostic parts is task-dependent. Within the current Shanghai surveillance context, the reduced-view configurations identified for both Calyptratae and Culicidae appear sufficient for routine identification purposes. However, should the framework be extended to broader surveillance scenarios involving a larger number of species or greater morphological similarity, the relative importance of individual anatomical parts, as well as the size and composition of the minimal diagnostic subset, may vary accordingly.

### 4.3. Practical Robustness and Future Extension

The datasets were generated under conventional CDC collection and simple specimen-preparation procedures, and the images were acquired using routine microscopes from different regional units with varying configurations, including differences in color temperature and image resolution (Table A3). Before being analyzed by MVP-Net, the images were also subjected to data augmentation, including illumination-related perturbation and occlusion simulation (Figure A1). Under these conditions, useful classification performance was still maintained after resizing the inputs to 224 × 224, suggesting some potential for extension to less specialized imaging hardware, although this would still require dedicated validation under new acquisition conditions.

The present study focused on Pareto optimization of minimal view subsets for species identification within the collected taxa and did not incorporate sex as a prediction target. This remains an important limitation for vector surveillance, because biological sex is directly related to epidemiological significance. Future work should therefore examine whether sex recognition accuracy can be incorporated as an additional objective into the current Pareto-optimization framework, so that species identification, sex recognition, and minimal view selection can be jointly optimized.

## 5. Conclusions

This study evaluated whether multi-view morphological identification of mosquito and fly taxa under routine surveillance in Shanghai could be simplified while preserving comparable classification performance. Using microscopy-based multi-view datasets of Calyptratae and Culicidae collected through approximately one year of routine surveillance by the Shanghai CDC system, MVP-Net identified compact subsets of diagnostically informative anatomical views while reducing image acquisition burden and computational cost.

For the 12-species Calyptratae dataset, the recommended configuration reduced the required input from 8 views to 5, lowered computational cost by 37.49%, and maintained 86.11% accuracy, remaining within 0.93% of the all-view configuration. For the 5-species Culicidae dataset, the required input was reduced from 11 views to 2, with an 81.82% reduction in computational cost while maintaining 100% accuracy for the species included in the current dataset. These findings indicate that, within the taxa routinely monitored in Shanghai, comparable identification performance can be achieved using a reduced set of anatomical views, although the number and composition of the selected views vary according to the specific taxonomic task.

Overall, these results support the application of MVP-Net as a regional auxiliary identification framework for mosquito and fly taxa under routine surveillance in Shanghai. Rather than minimizing the number of views at any cost, the framework provides a way to identify compact view subsets that preserve most of the discriminative information needed for identification under the current dataset setting. Future work should expand the regional and taxonomic coverage of the datasets and examine whether sex recognition accuracy can be incorporated as an additional objective into the current Pareto-optimization framework.

## Figures and Tables

**Table 1:** Quantitative analysis of Pareto-optimization strategies for Calyptratae.

λ	Number of Parts	Accuracy (%)	Accuracy Drop (%)	FLOPs (G)	FLOPs Saved (%)	Configuration
0.1, 0.2	8	87.04	0.00	11.31	0.00	All-view
0.3	7	87.04	0.00	9.90	12.47	Reduced
0.4	5	86.11	0.93	7.07	37.49	Recommended
0.5	3	84.26	2.78	4.24	62.51	Aggressive
0.6	1	83.33	3.71	1.41	87.53	Single-view

Note: For clarity, the table uses “all-view” to denote the configuration using all captured morphological views, “reduced” to denote intermediate Pareto-derived settings, “recommended” to denote the preferred operating point highlighted in the main analysis, and “aggressive” to denote the more strongly compressed setting with a clearer accuracy trade-off.

**Table 2:** Quantitative analysis of Pareto-optimization strategies for Culicidae.

λ	Number of Parts	Accuracy (%)	Accuracy Drop (%)	FLOPs (G)	FLOPs Saved (%)	Configuration
0.00	11	100.00	0.00	49.56	0.00	All-view
0.05	9	100.00	0.00	40.55	18.20	Reduced
0.10	8	100.00	0.00	36.04	27.28	Reduced
0.15	3	100.00	0.00	13.52	72.72	Reduced
0.20	2	100.00	0.00	9.01	81.82	Recommended
0.25	1	97.18	2.82	4.50	87.53	Single-view

## Data Availability

The data presented in this study are available on request from the corresponding author.

## Abbreviations

The following abbreviations are used in this manuscript:

FLOPs	Floating point operations
DL	Deep learning
CNNs	Convolutional neural networks
ViTs	Vision transformers
MOO	Multi-objective optimization
PE	Positional encoding
CE	Cross-entropy
GUI	Graphical user interface
CDC	Center for Disease Control and Prevention
CSV	Comma-separated values

## Appendix A

**Table A1:** District and month of the Calyptratae species included in the Shanghai routine surveillance dataset.

Species	District Coverage	Month Coverage	Total
*Aldrichina grahami*	7	10	39
*Boettcherisca peregrina*	8	7	58
*Calliphora nigribarbis*	2	2	11
*Chrysomya megacephala*	8	7	55
*Fannia prisca*	6	7	22
*Lucilia cuprina*	6	8	37
*Lucilia illustris*	8	10	38
*Lucilia sericata*	8	7	62
*Muscina angustifrons*	7	8	40
*Musca domestica*	8	7	67
*Musca sorbens*	8	7	32
*Muscina stabulans*	8	8	51
Total	8	10	512

Note: District coverage indicates the number of administrative districts in Shanghai from which each species was collected, and month coverage indicates the number of collection months represented in the dataset.

**Table A2:** Dataset split distribution of Calyptratae species.

Species	Training	Validation	Total
*Aldrichina grahami*	31	8	39
*Boettcherisca peregrina*	46	12	58
*Calliphora nigribarbis*	8	3	11
*Chrysomya megacephala*	44	11	55
*Fannia prisca*	17	5	22
*Lucilia cuprina*	29	8	37
*Lucilia illustris*	30	8	38
*Lucilia sericata*	49	13	62
*Muscina angustifrons*	32	8	40
*Musca domestica*	53	14	67
*Musca sorbens*	25	7	32
*Muscina stabulans*	40	11	51
Total	404	108	512

**Table A3:** Microscope specifications across representative geographic collection regions in Shanghai city.

Site	Microscope Model	Sensor	Resolution (*W* × *H*/MP)	Color Temperature
Putuo	Leica SAPO (Leica, Wetzlar, Germany)	Leica Flexacam C1 (Leica, Wetzlar, Germany)	3840 **×** 2160/8.3 MP	5500 K
Qingpu	Leica S9i (Leica, Wetzlar, Germany)	Integrated S9i (Leica, Wetzlar, Germany)	3648 **×** 2736/10.0 MP	4500 K
Fengxian	Olympus SZ61 (Olympus, Tokyo, Japan)	External CMOS (Olympus, Tokyo, Japan)	2592 **×** 1944/5.0 MP	Variable
Customs	Olympus CX31 (Olympus, Tokyo, Japan)	Nikon D300 (Nikon, Tokyo, Japan)	4288 **×** 2848/12.3 MP	3200–3400 K

## Appendix B

**Table A4:** District and month of the Culicidae species included in the Shanghai routine surveillance dataset.

Species	District Coverage	Month Coverage	Total
*Culex tritaeniorhynchus*	8	5	79
*Anopheles sinensis*	6	6	77
*Culex pipiens pallens*	8	5	70
*Aedes albopictus*	8	4	70
*Armigeres subalbatus*	8	5	67
Total	8	8	363

**Table A5:** Dataset split distribution of Culicidae species.

Species	Training	Validation	Total
*Culex tritaeniorhynchus*	63	16	79
*Anopheles sinensis*	61	16	77
*Culex pipiens pallens*	56	14	70
*Aedes albopictus*	56	14	70
*Armigeres subalbatus*	53	14	67
Total	289	74	363

## Appendix C

To comprehensively evaluate the performance of different view selection strategies and the Pareto-optimized configurations, five multi-dimensional metrics are defined. These metrics, visualized in the radar charts (Figure 4D and Figure 6C), quantify the trade-off between taxonomic accuracy and computational efficiency.

The following variables are used in the definitions: $Acc_{subset}$ and $Acc_{base}$ denote the Top-1 accuracy of the selected subset and the all-view baseline, respectively; $Acc_{single}$ represents the accuracy of the best-performing single-view model; $N_{subset}$ and $N_{total}$ are the number of selected views and the total available views; $FLOPs_{subset}$ and $FLOPs_{total}$ refer to the computational costs of the subset and the baseline.

1. Accuracy preservation (*AP*)

Measures the extent to which the optimized subset maintains the performance of the all-view baseline:

$$AP=\frac{Acc_{subset}}{Acc_{base}}$$

2. Marginal contribution (*MC*)

Quantifies the average accuracy gain per selected part relative to the most discriminative single view:

$$MC=\frac{Acc_{subset}-Acc_{single}}{N_{subset}}$$

3. Resource efficiency (*RE*)

Evaluates the classification performance achieved per unit of computational cost:

$$RE=\frac{Acc_{subset}}{FLOPs_{subset}}$$

4. Part reduction (*PR*)

Represents the proportion of redundant morphological views eliminated from the input set:

$$PR=1-\frac{N_{subset}}{N_{total}}$$

5. Deployment advantage (*DA*)

A composite index measuring the synergy between computational savings and accuracy retention, reflecting the model’s suitability for resource-constrained edge devices:

$$DA=\left(1-\frac{FLOPs_{subset}}{FLOPs_{total}}\right)\times \frac{Acc_{subset}}{Acc_{base}}$$
